# Assessment of toothbrushing, bleaching pen and bleaching mouthwash in removing stains from tooth structure and single-shade resin composite

**DOI:** 10.1038/s41598-026-39375-w

**Published:** 2026-03-03

**Authors:** Tamer M. Hamdy, Zeinab M. Zaki, Rasha M. Abdelraouf

**Affiliations:** 1 Restorative and Dental Materials Department, Oral and Dental Research Institute, National Research Centre (NRC), Dokki, Giza, 12622 Egypt; 2 Biomaterials Department, Faculty of Dentistry, Cairo University, Cairo, 11553 Egypt

**Keywords:** Bleaching pen, Bleaching mouthwash, Toothbrushing, Color changes, Coffee staining, Single shade resin composite, Vita easy shade spectrophotometer, Shade guide units (SGU) and color difference (∆E_00_), Dental materials, Restorative dentistry

## Abstract

The purpose of this study was to evaluate the ability of bleaching pen and bleaching mouthwash to remove coffee stains from teeth surfaces and single-shade resin composite restorations by assessing their Vita Classic shades, changes in the Shade Guide Units (ΔSGU) and color differences (∆E00). In addition, a control group subjected to simulated toothbrushing with non-whitening toothpaste was included for comparison. Class V cavities were prepared in the labial surfaces of 30 extracted sound anterior teeth and restored with single-shade resin composite. The restored teeth were immersed in coffee at 37 °C for 12 days. The stained restored teeth were randomly distributed into three groups (n = 10): Group 1: teeth subjected to simulated toothbrushing with non-whitening toothpaste for one week (control), while groups 2 and 3 were divided according to the bleaching system applied for one week as follows: Group 2: bleaching pen and Group 3: bleaching mouthwash. Using a spectrophotometer (Vita Easy Shade V, Germany), the color was measured before and after coffee staining as well as after toothbrushing or bleaching in each tooth at the middle 1/3 (tooth surface) and cervical 1/3 (restoration). The assessment was performed in two approaches: (a) Vita Classic shades which were used to calculate ΔSGU (b) Color parameters which were utilized to assess the color difference (ΔE00). After coffee staining, the color changes were higher in the teeth surfaces (ΔSGU = 7 and ΔE00=23.8) than restorations (ΔSGU = 4 and ΔE00=8.3). These color changes were unacceptable in both the teeth and resin composite restorations (exceeding the acceptability threshold; ΔE00=1.8). The effect of toothbrushing in removing coffee stains was significantly lower than the bleaching pen and the bleaching mouthwash in both resin composite restorations and teeth (least ΔE00, P = 0.0001). After toothbrushing, the shade of the stained restorations did not change than that after coffee (ΔSGU = 0), while the shade of the stained teeth became slight lighter but still darker than the baseline. After applying the bleaching pen or the bleaching mouthwash, the color returned to the baseline in both the restorations and teeth (ΔSGU = 0). Using the ΔE00 to compare the stain removal potentials of both bleaching pen and bleaching mouthwash, there were no significant differences between them in both resin composite restorations and teeth at cervical and middle one-thirds (P = 0.1 and 0.2, respectively). Toothbrushing with non-whitening toothpaste partially reduced coffee stains, but not completely. In contrast, bleaching pens and bleaching mouthwashes effectively removed coffee discoloration from both teeth and single-shade resin composite. Color changes could be assessed using the Vita Classic shade guide unit differences (ΔSGU) and color difference (ΔE₀₀).

## Introduction

Resin composite is a widely used direct aesthetic restorative material^1^. Several modifications have been conducted to improve its properties and facilitate the clinical procedure^2^. Single-shade resin composite was developed as an innovative smart material eliminating the need for a complex shade selection procedure^3^. The color of such composites could blend with the surrounding color of the natural tooth. Thus, mimicking the different color shades with just one single shade provides excellent color matching in minimum clinical steps^4^.

Beverages with staining potential, such as coffee, tea, red wine, and cola, are a significant cause of ageing and degradation of resin composites, leading to discoloration, staining, and surface changes^5^. These effects can compromise the esthetic and functional longevity of dental restorations^6–8^. The coffee has been identified as one of the causes of discoloring resin composite restorations. When resin composite restorations are frequently exposed to coffee, they can become stained, degraded, and ultimately show a noticeable color difference.

Previous research (2023) investigated the effect of coffee on the color of two single-shade resin composites *(Omnichroma and Venus Pearl)* versus those in distilled water. The coffee resulted in a significant unacceptable color change in both materials^9^. This was confirmed by another study in 2024, which investigated the color changes of the single-shade resin composite (Omnichroma) exposed to different stains from coffee and tea and then compared to artificial saliva. The coffee led to the highest staining^10^.

This discoloration can disappoint patients and may result in extra costs for replacing the filling^11^. In addition, the coffee could stain the natural teeth surfaces^12^. Thus, it is essential to restore the color of the stained teeth and restorations.

Dental bleaching products could enhance the esthetics of the discolored teeth and resin composite restorations. In-office and at-home bleaching products mainly differ in the type and concentration of the bleaching agent. In-office systems use high concentrations of hydrogen peroxide (15% to 45%) or carbamide peroxides (≥ 37%), whereas at-home products contain lower concentrations of hydrogen peroxide (3–10%) or carbamide peroxide (10–22%)^13,14^.

It was reported previously that both in-office and at-home bleaching agents could adversely affect the surface properties of resin composites. However, in-office bleaching caused a greater increase in surface roughness and a more pronounced reduction in microhardness compared with at-home bleaching, due to higher peroxide concentrations^15^.

The mechanism of degradation was related to the oxidative action of hydrogen or carbamide peroxide, which generates free radicals that can degrade the organic resin matrix by breaking polymer chains, reducing cross-link density, softening the superficial layer, and weakening the filler–matrix interface, promoting filler debonding or loss^16^. An increase in surface roughness, reduction in microhardness of resin-based restorations, and teeth sensitivity have been reported following peroxide exposure with higher concentrations^15,17^.

Recently some at-home bleaching products utilize a reduced concentration of hydrogen peroxide (1–3%) to reduce the possible adverse effects^5^. Among these products are bleaching pens and bleaching mouthwash, which could preserve the surface integrity, especially with short-term use, and remove stains, restoring esthetics to both restorations and natural teeth^18^.

Color measurement is crucial for evaluating successful restorations. The Vita Easy Shade spectrophotometer is widely used for color assessment of teeth and dental materials due to its reliable and reproducible results^19^. The measurement of the color changes could be achieved through different approaches. It could be calculated utilizing the CIEDE2000 color difference (∆E_00_), and comparing the resultant ∆E_00_ values to the perceptibility and acceptability threshold^19,20^. Moreover, the color changes could be expressed directly through Vita Classic shades^21^ with an additional informative step through using the “Shade Guide Units (SGU)” system. The change in “Shade Guide Units (ΔSGU)” was applied to provide a numerical representation of the color changes based on the Vita Classical shade guide.

In the SGU system, the Vita Classical shade guide is first re-organized into a linear scale based on lightness values (value-based order) rather than the manufacturer’s original hue/chroma grouping. The correct procedure is to organize shades according to the lightness values in decreasing order and then, they are given numbers from 1 to 16 as follows: B1(lightest = 1), A1, B2, D2, A2, C1, C2, D4, A3, D3, B3, A3.5, B4, C3, A4, and C4 (darkest = 16). The ΔSGU is calculated as follows (ΔSGU = post-treatment SGU - pre-treatment SGU). Negative ΔSGU denotes a lighter shade was obtained (tooth whitening), while positive ΔSGU represents a darker shade had occurred (tooth discoloration). This system is used to quantify the degree of color changes overtime or after treatments such as bleaching^22^.

There are limited data available about at-home bleaching products that use lower concentrations of hydrogen peroxide and the presence of conflicting results. Moreover, understanding the efficiency of single-shade resin composites in such situations is critical, especially upon using different color measurement approaches.

The aim of the current study was to assess the capability of the bleaching pen and bleaching mouthwash to remove the coffee stains from both the teeth and single shade resin composite restorations by evaluating their Vita Classic shades, changes in the Shade Guide Units (ΔSGU) and color differences (∆E_00_). A control group subjected to simulated toothbrushing with non-whitening toothpaste was added for comparison.

The null hypothesis postulated that there would be no differences in the staining removal potential of the toothbrushing, bleaching pen and bleaching mouthwash in results represented by the Vita Classic shades and changes in the Shade Guide Units (ΔSGU) as well as the color difference (∆E_00_).

## Methods

This in vitro study was approved by the Medical Research Ethical Committee (MREC) of the National Research Centre (NRC), Cairo, Egypt (Reference number: 0118082022). All patients provided informed consent prior to the extraction procedures. Two different at-home bleaching methods were evaluated (Colgate Optic Whitening Pen and Colgate Optic White, Whitening Mouthwash). One type of single-shade resin composite (Omnichroma, Tokuyama Dental, Tokyo, Japan) was used to restore the prepared class V cavities in the extracted incisors. Materials used in the study are displayed in Table 1.

**Table 1:** Materials used in the study.

Materials	Ingredient	Manufacturer
Single-shade resin composite	Fillers: Uniform sized supra-nano spherical silica-zirconia filler loading 79 wt% (68 vol %). Matrix: UDMA, TEGDMA.	Omnichroma (Tokuyama Dental, Taitouku, Tokyo, Japan).
Colgate Optic Whitening Pen	3% Hydrogen peroxide, acrylates/octylacrylamide copolymer, alcohol, water.	Colgate Optic White Overnight Teeth Whitening Pen, Colgate, NY, USA.
Colgate Optic White, Whitening Mouthwash	Water, glycerin, propylene glycol, sorbitol, 1–2% hydrogen peroxide, polysorbate 20, sodium acrylates/methacryloylethyl phosphate copolymer, phosphoric acid, citric acid, flavor, PVM/MA copolymer, sodium saccharin.	Colgate-Palmolive Co., NY, USA.

### Sample size calculation

The sample size calculation was determined on a similar study^23,24^. With an alpha level of 0.05, an effect size of 0.25, and a power of 90%, a sample size was calculated using G*Power software version 3.1.9.7 (Heinrich Heine University Duesseldorf, Duesseldorf, Germany). The estimated sample size was 10 specimens per group.

### Study design

**Fig. 1:**
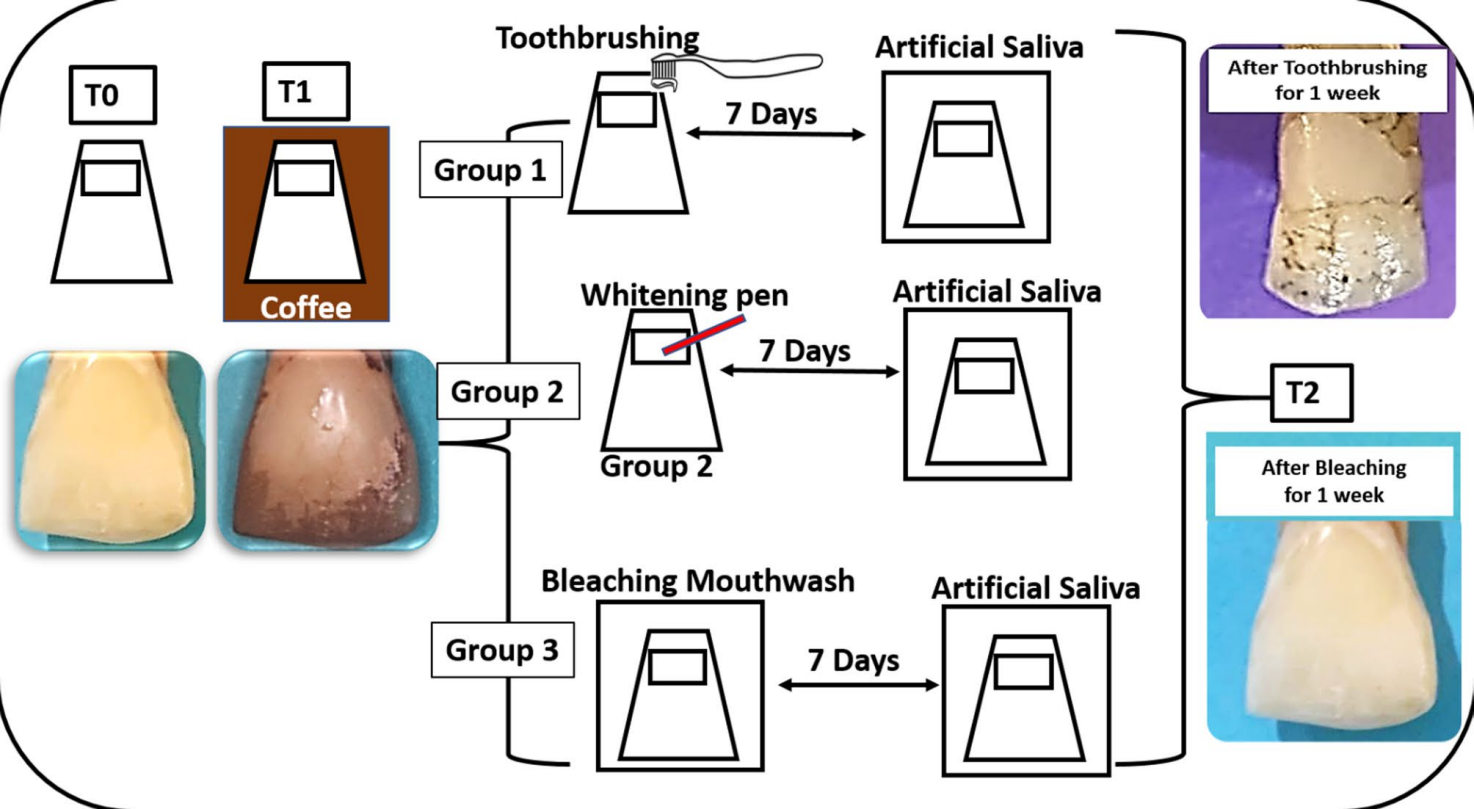
Diagram representing the study design.

Figure 1 illustrates the study design. Standardized class V cavities were prepared in twenty sound freshly extracted human anterior teeth and restored by single-shade resin composite (T0). The restored teeth were stained by coffee (T1). The stained teeth were randomly distributed into three groups (*n* = 10), according to the method applied for one week to remove the coffee stains: Group 1: Simulated toothbrushing with non-whitening toothpaste, Group 2: Bleaching pen, and Group 3: Bleaching mouthwash (T2).

The color parameters were measured for both the restorations at the cervical 1/3 and the teeth structure at the middle 1/3. The color measurements were performed at three-time intervals: Before staining (T0), after coffee staining (T1) and after bleaching for one week (T2) using a spectrophotometer (Vita Easy Shade, Vita Zahnfabrik, Bad Säckingen, Germany). The color of both the restorations and the teeth was described by the Vita Classic shades using the Vita Easy Shade spectrophotometer at the cervical and middle 1/3, respectively, at the various time intervals. Changes in the “Shade Guide Units” (ΔSGU) and the color differences (∆E_00_) were calculated after staining as well as after bleaching.

### Teeth selection

Thirty sound freshly extracted anterior teeth were obtained, cleaned with periodontal curettes, and polished using pumice and rubber cups. For standardization, the selected teeth were chosen to have shade A3 according to the Vita Classic shade system determined by the Vita Easy Shade spectrophotometer (as an inclusion criterion in teeth selection).

### Cavity design

In each tooth, a standardized class V cavity (4 mm width x 4 mm height x 2 mm depth) was prepared 2 mm above the cemento-enamel junction (CEJ). The cavities were performed on the buccal cervical part of the teeth using water-cooled round-shaped diamond bur (Komet Dental, Lemgo, Germany) following the previously published protocol^25^.

### Resin composite application

The single shade resin composite (Omnichroma, Tokuyama Dental, Japan) was applied following the manufacturer’s instructions. All procedures were performed by the same operator, with a standardized application time monitored using a stopwatch.

An acid etching (Eco-Etch gel, Ivoclar Vivadent, Liechtenstein) was applied for 15 s, followed by washing and gentle air-drying using an air-water syringe. A bonding agent (Single Bond Universal, 3 M ESPE, USA) was applied using a thin-brush in a rubbing motion for 20 s according to the manufacturer’s instruction; afterwards, the teeth were air-dried for 5 s and followed by light curing for 10 s using a (LED) curing unit (Mini-LED, Satelec, Acteon, France) with a wavelength of 400–500 nm and a power density of 1000 mW/cm^2^. The resin composite was applied to fill the prepared cavity and light-cured for 40 s. Finishing and polishing of resin composite was performed using a special kit (Shofu Composite Polishing Kit, Shofu Dental GmbH, Ratingen, Germany).

### Color measurement before staining (T0)

The color was measured for both the restorations at the cervical 1/3 and the teeth at the middle 1/3, respectively, using an oral spectrophotometer (Vita Easy Shade V, Vita Zahnfabrik, Bad Säckingen, Germany). This model had a small tip (~ 4 mm) for more precise measurement on limited surfaces, such as cervical. The color measurement was performed using two approaches: (a) correlation to Vita Classic shades and (b) assessment of color parameters.

**Fig. 2:**
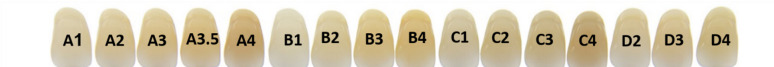
Vita Classical Shades detected by Vita Easy Shade Spectrophotometer.

The color of both the restorations and teeth was correlated to the Vita Classic shades before staining (Baseline). These shades included 4 groups (A, B, C and D): A (reddish-brownish), B (reddish-yellowish), C (greyish shades) and D (reddish-grey). In each group, there is a subclassification from the lightest to the darkest, where “1” is the lightest shade while “4” is the darkest shade: A1-A4, B1-B4, C1-C4 and D2-D4, Fig. 2. These shades were used to calculate the changes in the “Shade Guide Units” (ΔSGU), as would be clarified later.

For each shade, the following color parameters were recorded: a, b and L (where a = hue across the red-green axis, b = hue across the yellow-blue axis and L = lightness). These parameters were used to assess the color difference (∆E_00_), as would be described later.

### Staining procedure

Coffee was used as the staining medium for the restored teeth. The solution was prepared by dissolving 3.6 g of coffee powder (Nescafé Classic; Nestlé, Switzerland) in 300 millilitres of boiling distilled water, stirring for 10 min, and then filtering the mixture through filter paper^26^. The restored teeth were immersed in coffee at 37 °C for 12 days, resembling one year clinically (as one day of immersion resembles one month intra-orally)^27–29^.

### Color measurement after staining (T1)

After staining, Vita Classic shades and the color parameters were measured for both the restorations at the cervical 1/3 and the teeth structure at the middle 1/3 using the Vita Easy Shade spectrophotometer.

### Calculation of shade guide units changes (ΔSGU) after staining

The obtained Vita Classical shades by the Vita Easy Shade spectrophotometer were given specific numbers according to the SGU system for both the teeth and restorations before and after coffee staining.

These numbers are from 1 to 16, given to the tabs of the Vita Classic shade guide arranged from the lightest to the darkest as follows:1 = B1 (lightest), 2 = A1, 3 = B2, 4 = D2, 5 = A2, 6 = C1, 7 = C2, 8 = D4, 9 = A3, 10 = D3, 11 = B3, 12 = A3.5, 13 = B4, 14 = C3, 15 = A4, and 16 = C4 (darkest).

The difference in Shade Guide Units (ΔSGU) is calculated as ΔSGU = post-treatment SGU - pre-treatment SGU. In this case the ΔSGU = SGU_T1_ - SGU_T0_, where SGU_T1_denoted the numerical SGU value of the Vita Classic shade after coffee staining for the teeth or the restorations, while SGU_T0_ was that for the baseline.

Positive results indicate a discoloration and darkening effect, while negative results denote more lightness and a whitening effect.

### Color difference (ΔE_00_) assessment after staining

The color changes (ΔE_00_) after staining (T1) were compared to that original color before staining (T0). The color difference was calculated according to “La Commission Internationale de l’Éclairage” using the CIEDE2000 formula^28,30^:

$$\:\varDelta\:\mathrm{E}\mathrm{00}=\sqrt{{\left(\frac{\stackrel{\prime }{{\Delta\:}\mathrm{L}}}{\mathrm{k}\mathrm{L}\bullet\:\mathrm{S}\mathrm{L}}\right)}^{2}+{\left(\frac{\stackrel{\prime }{{\Delta\:}\mathrm{C}}}{\mathrm{k}\mathrm{c}\bullet\:\mathrm{S}\mathrm{c}}\right)}^{2}+{\left(\frac{\stackrel{\prime }{{\Delta\:}\mathrm{H}}}{\mathrm{k}\mathrm{H}\bullet\:\mathrm{S}\mathrm{H}}\right)}^{2}+\left(\mathrm{R}\mathrm{T}\left(\frac{\stackrel{\prime }{\varDelta\:C}}{k\mathrm{C}\bullet\:S\mathrm{C}}\right)\left(\frac{\stackrel{\prime }{{\Delta\:}\mathrm{H}}}{\mathrm{k}\mathrm{H}\bullet\:\mathrm{S}\mathrm{H}}\right)\right)}$$

where ΔL = the lightness difference, ΔC = the chroma difference, ΔH = the hue difference, and k_L_, S_L_, k_C_, S_C_, k_H_, and S_H_ are the constants of the coefficient.

The resultant ∆E_00_ values were compared to the perceptibility threshold, which represents the first visually detectable color variation (∆E_00_ = 0.8), and the acceptability threshold, which represents the starting point of unacceptable color change (∆E_00_ = 1.8)^30,31^.

### Grouping according to toothbrushing (Control) and bleaching methods

The stained restored teeth were randomly distributed into three groups (*n* = 10): Group 1: teeth subjected to simulated tooth brushing with non-whitening toothpaste for one week, while groups 2 and 3 were divided according to the bleaching system applied for one week as follows: Group 2: bleaching pen and Group 3: bleaching mouthwash.

### Group 1: control

After staining, the teeth in the control group were subjected to simulated brushing using a non-whitening toothpaste (Signal 2, Unilever, Egypt). Toothbrushing was performed using an automatic brushing machine with soft-bristled toothbrush heads under a load of 200 g. A slurry of non-whitening toothpaste and distilled water (1:2 w/w) was prepared and replenished as needed. Each tooth was brushed for 1,200 strokes per day, simulating two minutes of manual brushing twice daily, for 7 consecutive days. Between brushing sessions, the teeth were stored in artificial saliva at 37 °C to simulate intraoral conditions. After each brushing session, the teeth were rinsed with distilled water and gently air-dried. Color measurements were performed before and after staining as well as after the 7-day brushing to evaluate the effect of simulated toothbrushing on stain removal^32^.

### Group 2: bleaching pen

Clinically, the gel of the bleaching pen is applied on the labial surface of the teeth using its applicator tip. It is painted evenly to form a thin coating. The material is left undisturbed for a specified period (≈ 60 s) to allow it to dry and adhere, creating a visible film. This film remains in place overnight (≈ 8 h). In the next morning, it is removed mechanically by peeling or brushing, then rinsing.

In this study, to simulate the clinical condition, the gel of the bleaching pen was applied to the buccal surface of the restored teeth until a visible layer was created, left to dry for 60 s, then left upon restored teeth surfaces for eight hours and stored in artificial saliva (Sigma-Aldrich CO, MO, USA) to simulate exposure overnight intra-orally^33^. The coat was then peeled from the specimen’s surface by toothbrush after 8 h and stored in the artificial saliva for the rest of the day (16 h)^33^. This cycle was repeated for seven days.

### Group 3: bleaching mouthwash

For the third group, each tooth was immersed in 20 ml of bleaching mouthwash for 60 s twice a day, resembling rinsing in the morning and at night^27^. For the rest of the day, the teeth were left in the artificial saliva simulating the clinical condition after spitting out the bleaching mouthwash^27^. That was repeated for seven days.

### Reassessment of color and resultant changes (ΔSGU and ΔE_00_) after bleaching (T2)

After toothbrushing and bleaching (either by pen or mouthwash), the shades and the color parameters were measured by the Vita Easy Shade spectrophotometer (T2) for the teeth and the restorations. The ΔSGU and ΔE_00_ were calculated by comparing the SGU as well as the color parameters after bleaching versus that before bleaching for each tooth and the restoration, as previously discussed.

### Statistical analysis

The Statistical Package for the Social Sciences (SPSS, IBM, New York, NY, USA) was used to statistically analyse the data. The data was described using the mean and standard deviation, and the Shapiro-Wilk and Kolmogorov-Smirnov tests revealed a normal distribution. The significance level was set at *p* ≤ 0.05. One-way ANOVA and post hoc tests were used to compare the mean color differences values (ΔE_00_) between the toothbrushing and two bleaching methods measured at the middle and cervical one-thirds of the teeth and restoration surfaces, respectively.

## Results

The baseline of the extracted anterior teeth before staining was “A3” (an inclusion criterion in teeth selection), where group “A” denoted reddish-brownish shades. After coffee staining, the color was shifted to “C4”, where the C group referred to a greyish shade and 4 denoted the darkest shade in this group. After the simulated tooth brushing, the shade of the stained teeth became slightly lighter (shade “A4”) yet darker than the baseline (shade “A3”), Table 2. After the whitening effect by bleaching pen or bleaching mouthwash, the color returned to the baseline (shade “A3”), Table 2.

While the baseline of the resin composite restorations before staining was “A2” measured at the cervical one-third, where group “A” denoted reddish-brownish shades. After coffee staining, their color was still in the “A” group but was shifted to “A3”, which was darker than the baseline. After the simulated tooth brushing, the shade of the stained restorations did not change (still shade “A3”), Table 2. After the whitening effect by bleaching pen or bleaching mouthwash, the color returned to the baseline (shade “A2”), Table 2.

**Table 2:** The vita classic shades were measured for the restorations and the teeth.

Vita Classic shades	Baseline (T0) (Before Staining)	After Coffee Staining (T1)	Bleaching Method	After Bleaching for 7 days (T2)
Restoration at cervical 1/3	A2	A3	Toothbrushing	A3
			Bleaching Pen	A2
			Bleaching Mouthwash	A2
Tooth at middle 1/3	A3	C4	Toothbrushing	A4
			Bleaching Pen	A3
			Bleaching Mouthwash	A3

### Shade guide unit difference (ΔSGU)

Regarding the changes in the Shade Guide Units (ΔSGU) after staining in the restorations, it was 4 (A3-A2 = 9 − 5). As for the teeth, it was 7 (C4-A3 = 16 − 9), indicating more discoloration.

The effect of toothbrushing in removing coffee stains had a representative image in Fig. 1. After toothbrushing the stained restorations (T2), the shade when compared to the baseline (T0), the calculated ΔSGU for the restorations was 4 (A3-A2 = 9 − 5), indicating discoloration. Also, in the teeth, the ΔSGU was 6 (A4-A3 = 15 − 9), revealing discoloration as well. Comparing the ΔSGU after toothbrushing (T2) versus that after coffee staining (T1), it was zero for the restorations, indicating no color change and persistence of the coffee stains, and that for the teeth was − 1 (A4-C4 = 15–16), representing a slight reduction in the staining, yet still darker than the baseline.

After bleaching (T2), the ΔSGU was zero compared to the baseline (T0), as the color returned to the same original shade in both the teeth and restorations. Comparing the ΔSGU after bleaching (T2) versus that after coffee staining (T1), it was − 4 (A2-A3 = 5–9) and − 7 (A3-C4 = 9–16) for the restorations and the teeth, respectively.

### Color difference (ΔE_00_)

Concerning the calculated color differences (ΔE_00_), after coffee staining, there were unacceptable color changes in both resin composite restorations and teeth at the cervical and middle one-thirds (ΔE_00_=8.3 and 23.8), respectively (Table 3), i.e., above the acceptability threshold (ΔE_00_=1.8). The color changes in teeth (ΔE_00_=23.8) were significantly higher than the resin composite restorations (ΔE_00_=8.3).

**Table 3:** The color differences (ΔE_00_) after staining (Difference between T1 and T0).

ΔE_00_ before and after staining	ΔE_00_	Compared to the Acceptability threshold
Restorations at cervical 1/3	8.3	Above acceptability threshold
Teeth at middle 1/3	23.8	
P value	0.0001*	

The effect of toothbrushing in removing coffee stains was significantly lower than the bleaching pen and bleaching mouthwash in both resin composite restorations and teeth at the cervical and middle one-thirds (*P* = 0.0001) (Table 4). The toothbrushing effect led to significantly higher color changes in stained teeth than in the stained resin composite restorations (*P* = 0.0001).

**Table 4:** The color differences (ΔE_00_) after toothbrushing or bleaching (Difference between T2 and T1).

ΔE_00_ before and after bleaching	Tooth Brushing ΔE_00_	Bleaching Pen ΔE_00_	Bleaching Mouthwash ΔE_00_	*P* value	Compared to Acceptability threshold
Restorations at cervical 1/3	1.1^aA^	4.9^bA^	4.5^bA^	0.0001*	Above acceptability threshold except the toothbrushing with restorations (minimal color change)
Teeth at middle 1/3	8.8^aB^	25^bB^	25.9^bB^	0.0001*	
P value	0.0001*	0.0001*	0.0001*		

*Significant difference; significant (P < 0.05). Different small letters in the same row indicated a significant difference. Different capital letters in the same column indicated a significant difference.

**Fig. 3:**
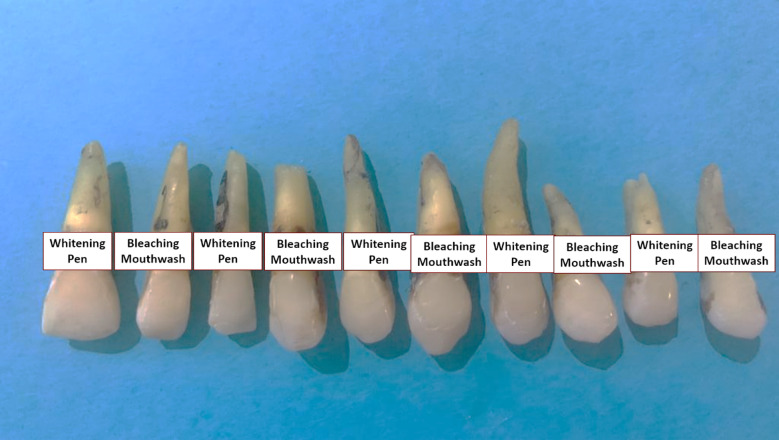
Extracted teeth after bleaching by either bleaching pen or bleaching mouthwash to remove coffee stains.

Comparing the stain removal potentials of both bleaching pen and bleaching mouthwash, there were no significant differences between them in both resin composite restorations and teeth at the cervical and middle one-thirds (*P* = 0.1 and 0.2, respectively) (Table 4; Fig. 3). The bleaching effect led to significantly higher color changes in stained teeth than in the stained resin composite restorations (*P* = 0.0001).

## Discussion

Comparing the effectiveness of conventional toothbrushing, the bleaching pen, and the bleaching mouthwash in removing the coffee stains from both restorations and teeth surfaces, the null hypothesis was rejected, as the effect of the toothbrushing was significantly lower than the two bleaching methods, with no significant difference between them.

In this study, the extracted teeth were stored at 37 °C, simulating mouth temperature, for 12 days, as this time period mimics a one-year (12-month) period in a real-life clinical setting^34,35^. The shade of the extracted anterior teeth was selected to be “A3” as an inclusion criterion, as it was one of the most common shades of natural teeth, especially in females^36^.

After restoring the prepared cavities in the shade of the resin composite restorations, “A2” was a little bit different than tooth “A3”. It was reported that the universal shade resin composite’s color matching to the patients’ teeth was satisfactory but not perfect^37^.

After coffee staining, the color of teeth was shifted from “A3” to “C4” (the darkest greyish shade). There were two main reasons that could explain coffee’s teeth-staining potential^38^. The first reason was its tannin content (a type of polyphenol that breaks down in water). Tannins can bind to the teeth surfaces, allowing color compounds to adhere and cause stains. The second cause is the coffee acidity, which can soften tooth enamel.

The color of the resin composite restorations after coffee staining was still in the “A” group but was shifted from “A2” to “A3”, which was darker than the baseline. This discoloration was explained previously by the coffee tannins.

This was also confirmed by the calculated color differences (ΔE_00_), which showed unaccepted color changes (i.e., above the acceptability threshold, ΔE_00_=1.8) in both resin composite restorations and teeth (ΔE_00_=8.3 and 23.8, respectively).

It was detected that the discoloration of the restorations was lower than the teeth structure. That was confirmed by the lower ΔSGU and ΔE_00_ values in the restorations (ΔSGU = 4 and ΔE_00_=8.3) than the teeth (ΔSGU = 7 and ΔE_00_=23.8) after staining. The difference between their chemical composition and structure may affect their response to the coffee and its acidity, which had been reported previously in the literature (pH ≈ 4.8–5.1)^39^. Tooth enamel was composed mainly of hydroxyapatite, a crystalline form of calcium phosphate which started to demineralize at a pH of about 5.5 or lower, leaving a rough surface with high stain liability^40^. While the resin composite was composed of filled resin-based materials. These fillers in the single-shade resin composite were 79 wt%, which were less reactive to acids than enamel^41^. Although the resin composites can be subjected to wear, and the long-term acid exposure can soften the resin matrix, leading to surface degradation or roughness, it was yet still less severe than that with natural enamel.

It was observed that there were higher deposited coffee stains at the incisal edge of the teeth than the rest of the teeth (Fig. 1). This variation may be due to the extraction process, which could lead to microcracks at the thin incisal edges.

Toothbrushing resulted in partial reduction of coffee stains from teeth surfaces, rather than complete elimination (Fig. 1). This may be explained by the penetration of coffee chromogens, such as tannins and polyphenols, into the enamel micro-porosities^42^. The conventional non-whitening toothpaste has limited abrasiveness, allowing removal of superficial deposits while leaving deeply retained pigments. Therefore, mechanical toothbrushing alone is insufficient to achieve complete stain elimination from teeth. The results of the current study agreed with a former one conducted on bovine labial enamel and concluded that that toothbrushing produced only limited removal of extrinsic stains and was ineffective against deeper discoloration^42^.

Regarding the resin composite restorations, the toothbrushing was not able to reduce the discoloration and the shade persisted as that after coffee. This can be attributed to the resin matrix which may undergo water sorption, allowing the coffee chromogens to diffuse into the subsurface and become entrapped within the polymer network rather than remaining on the surface^43^. Therefore, conventional toothpaste was insufficient to remove these internally absorbed stains. This agreed with a previous research which used the same single resin composite (Omnichroma) which highlighted the limited effect of mechanical toothbrushing on the resin’s surface^44^.

After applying the bleaching pen or bleaching mouthwash, the color returned to the baseline in both the restorations and the teeth (ΔSGU = 0). This may be due to their hydrogen peroxide content as an active ingredient. These peroxides release oxygen molecules that break down chromogens (pigmented compounds in stains). The chemical reaction oxidizes the organic molecules, making them colorless or less visible. As the shades reverted to the baseline after bleaching, there was no need to calculate the color difference (ΔE_00_) following bleaching versus the baseline.

Due to the higher deposited stains in the teeth than the resin composite restorations, the bleaching effect was more pronounced upon the teeth surfaces (ΔSGU = −7) than the restorations (ΔSGU = −4) despite both returned to the baseline.

No significant differences in the stain removal potentials of both bleaching pen and bleaching mouthwash, which may be due to the similarity in their active ingredient (hydrogen peroxide) with comparable concentration. However, the bleaching pens and bleaching mouthwashes could differ in their clinical application. The bleaching mouthwash, due to its liquid nature, has an easier application and generalized effect. On the other hand, the bleaching pen could be applied to a specific area or teeth due to its gel and resultant coat.

One of the limitations of this study was that one exposure time and one resin composite type were used. Another limitation is that the surface roughness of the restored teeth surfaces was not evaluated; thus, future investigations for surface roughness analysis would be valuable.

## Conclusions

Within the study’s limitations, it could be concluded that:

- Toothbrushing could partially reduce coffee stains but was ineffective in eliminating deeply penetrating stains.
- Bleaching pens and bleaching mouthwashes could remove coffee stains from both tooth surfaces and single-shade resin composite restorations.
- Dental color changes can be performed using the Vita Classic shade guide unit differences (ΔSGU) as well as color difference CIEDE2000 formula (ΔE₀₀).

### Significance

This in vitro study supports that conventional toothbrushing with non-whitening toothpaste can only partially reduce the extrinsic coffee stains. While the bleaching pens and bleaching mouthwash could effectively remove coffee stains from teeth and single-shade resin composite. Color could be assessed directly by Vita Classic shade guide unit differences (ΔSGU) or indirectly after calculating color differences (ΔE_00_).

## Data Availability

The data that support the findings of this study are available from the corresponding author upon reasonable request.
